# Prevalence of Methicillin-Resistant*Staphylococcus aureus* and Multidrug-Resistant Strains from Patients Attending the Referral Hospitals of Amhara Regional State, Ethiopia

**DOI:** 10.1155/2023/3848073

**Published:** 2023-06-20

**Authors:** Feleke Moges, Tadele Tamiru, Azanaw Amare, Getachew Mengistu, Setegn Eshetie, Mulat Dagnew, Tigist Feleke, Mucheye Gizachew, Wondwossen Abebe

**Affiliations:** ^1^Department of Medical Microbiology, School of Biomedical and Laboratory Sciences, College of Medicine and Health Sciences, University of Gondar, Gondar, Ethiopia; ^2^Institute of Biotechnology, College of Natural and Computational Sciences, University of Gondar, Gondar, Ethiopia; ^3^Department of Medical Microbiology, College of Health Sciences, Debre Markos University, Debre Markos, Ethiopia; ^4^Department of Hospital Laboratory, University of Gondar Comprehensive Specialized Hospital, Gondar, Ethiopia

## Abstract

**Background:**

*Staphylococcus aureus* (*S. aureus*) causes different types of human infections and can develop resistance to many antibiotics. There is a scarcity of data on the *mecA* gene and multidrug-resistant (MDR) strain distribution of this organism in developing countries, such as Ethiopia. This study investigated the presence of *mecA* gene and MDR profile of *S. aureus* among patients attending referral hospitals of Amhara regional state.

**Methods:**

Of the total of 110 isolates collected from Amhara regional referral hospitals, 70 MDR isolates were further processed for isolation of *S. aureus mecA* gene. Genomic DNA was isolated using a Sigma-Aldrich genomic DNA isolation kit for Gram-positive bacteria. Amplification of *S. aureus mecA* gene was performed with the amplicon size of 533 bp. Antimicrobial susceptibility test including methicillin resistance was determined by the Kirby–Bauer disc diffusion method.

**Results:**

The majority of the isolates were recovered from patients aged less than 5 years (51; 36.7%) and the least number of isolates was recorded in age group greater than 60 years (6; 4.3%). Most of the isolates were from blood (61; 43.9%), followed by wounds (45; 32.4%). A high resistance rate was observed in penicillin (81; 73.6%), followed by cotrimoxazole (78; 70.9%), ceftriaxone (76; 69%), erythromycin (66; 60%), and tetracycline (65; 59.1%). Phenotypically, considering cefoxitin as a surrogate marker, 38 (34.5%) of the isolates were methicillin-resistant. The overall MDR isolates were 80 (72.7%). The PCR amplification result of the *mecA* gene was 14 (20%). *Conclusions and Recommendations*. High rates of MDR and methicillin-resistant*S. aureus* were reported. PCR amplification indicated that 20% of MRSA isolates were the *mecA* gene carriers. Large-scale studies for the detection of MDR strains of *S. aureus* including MRSA using molecular techniques should be encouraged in the Amhara region.

## 1. Introduction

*Staphylococcus aureus* (*S. aureus*) is one of the most common causes of bacterial infection in humans that causes both community- and hospital-acquired infection of the skin, urinary tract, surgical site infections, osteomyelitis, septicaemia, and endocarditis [1]. *Staphylococcus aureus* has an extraordinary ability to develop resistance to many antibiotics. This was first revealed by the acquisition of *β*-lactamase on “penicillinase plasmids” and the subsequent response to *β*-lactamase stable derivatives by acquisition of staphylococcal cassette chromosome (SCCmec) elements by methicillin-resistant *S. aureus* (MRSA) [2].

Penicillin has been used as a drug of choice for *S. aureus* as it was discovered by Fleming in the 1940s, but with the widespread use of penicillin in the 1950s, penicillin-resistant *S. aureus* appeared in the hospitals [3, 4]. Penicillin-resistant*S. aureus* can produce penicillinase, which can hydrolyze the penicillin *β*-lactam ring, leading to resistance to penicillin. Later, scientists developed a new penicillinase-resistant semisynthetic penicillin named methicillin, which is resistant to the hydrolysis of *β*-lactamase [3, 5]. Therefore, in the widespread appearance of penicillin-resistant *S. aureus*, methicillin was used as a drug of choice for penicillin-resistant *S. aureus.* However, soon later MRSA strain was reported; this resistance was produced by a gene encoding the penicillin-binding protein 2a or 2′ (PBP2a or PBP2′) (*mecA*) which was integrated into the chromosomal element (SCCmec) of methicillin-sensitive *S. aureus* [6]. Available data show that the structural gene, *mecA*, is present in the resistant strains of *S. aureus*, but not in the susceptible ones [7]. This achievement has enabled the development of an alternative method for identifying methicillin-resistant *S. aureus* by detecting the *mecA* gene. Moreover, other than *macA* gene, *mecB, mecC*, and *macD* have been documented as responsible for methicillin resistance in the *Staphylococcaceae* family. The *mecB* and *mecD* genes were reported at first on the chromosome and/or on a plasmid of *Macrococcus caseolyticus*. Recently, the *mecB* gene was also documented on a plasmid of one MRSA isolated from a human patient [8].

In the present study, the polymerase chain reaction (PCR) was used to detect the methicillin resistance determinant by amplifying a 533-bp region of the *mecA* gene. The gold standard to determine MRSA genotypes is to detect conserved genes constantly found in the *mec*A gene, which is within the range of a particular chromosome in staphylococcal cassette chromosome (SCC*mec*) [9]. Therefore, amplification of *mec*A can be performed by using PCR, which is the gold standard for the detection of *mec*A gene [10]. No information on the distribution of the *mecA* gene on MRSA in Amhara region is available. Therefore, this study aimed at investigating the presence of *mecA* gene and multidrug-resistant (MDR) strain distribution of *S. aureus* among patients attending referral hospitals of Amhara regional state.

## 2. Materials and Methods

### 2.1. Bacterial Isolates

A total of 139 isolates of *S. aureus* were isolated between the periods of 2017 and 2018 from Amhara region referral hospitals (University of Gondar Comprehensive Specialized Hospital, Felege Hiwot Comprehensive Specialized Hospital, Dessie Referral Hospital, and Debre Markos Referral Hospital). Detailed data of sample size determination, sampling technique, and specimen collection were found from the previous two studies [11, 12]. All isolates were clinical isolates from different specimens such as blood, urine, wounds, discharges, and body fluids. Each clinical sample was cultured on mannitol salt agar and incubated at 37°C for 24 h. Further identification of *S. aureus* isolates was performed by colony morphology, Gram stain, and standard biochemical characteristics such as catalase, coagulase, and novobiocin susceptibility tests. The ATCC 25923 of *S. aureus* was used as reference strain.

### 2.2. Antibiotic Susceptibility Test

Susceptibility test was performed using the modified Kirby–Bauer disk diffusion method on Muller–Hinton agar following Clinical and Laboratory Standard Institute (CLSI) guidelines [13]. Pure colonies of freshly grown *S. aureus* suspension were prepared in 3–5 ml normal salineand turbidity was adjusted to 0.5 McFarland standards. The plates were allowed to dry for 3–5 minutes; then, discs were evenly distributed on the inoculated plate using sterile forceps and incubated at 37°C for 18–24 h. The susceptibility test for *S. aureus* was performed against erythromycin (ERY, 15 *μ*g), penicillin (PEN, I0 IU), clindamycin (CLI, 10 *μ*g), cotrimoxazole (SXT, 25 *μ*g), tetracycline (TET, 30 *μ*g), ciprofloxacin (CIP, 5 *μ*g), chloramphenicol (CHL, 30 *μ*g), gentamicin (GEN, 10 *μ*g), ceftriaxone (CRO, 30 *μ*g), and cefoxitin (FOX, 30 *μ*g) (all from Abtek bio.Ltd UK). Multidrug resistance patterns of the isolates were determined following the criteria set by Magiorakos et al. [14]. Using CLSI guidelines, the diameter of the zone of inhibition around the disc was measured and interpreted as sensitive, intermediate, and resistant.

### 2.3. Extraction of DNA from *S. aureus*

The clinical isolates were subcultured using nutrient agar medium and incubated for 24 h at 37°C. A single colony was taken from the previously subcultured medium and inoculated in to 10 ml Luria–Bertani (LB) broth medium, incubated at 37°C with a shaker incubator for 24 h. After 24 h incubation, genomic DNA was isolated using Sigma-Aldrich genomic DNA extraction kit for Gram-positive bacteria, and the isolation protocols were followed according to the manufacturer's instructions of Sigma-Aldrich. Finally, the extracted DNA was dissolved with Tris-EDTA buffer (10 mM Tris-Cl and 1 mM EDTA buffer), and the quality of isolated genomic DNA was confirmed by using NanoDrop and 1.5% agarose gel electrophoresis, and then it was stored at −21°C till use.

### 2.4. Amplification of *S. aureus mecA* Gene

MRSA isolates were identified by phenotypic method, and PCR (thermocycler machine) was performed to amplify the *S. aureus mecA* gene with the amplicon size of 533 bp using primers *mecA* forward sequence 5′-AAAATCGATGGTAAAGGTTGGC-3′ and *mecA* reverse sequence 5′-AGTTCTGGAGTACCGGATTTGC-3′ described in [15].

The specific oligonucleotide primers for *mec*A genes were diluted by using nuclease-free water according to the manufacture company information to get primary concentration equal to 100 pmol. Thermal cycler and the reaction mixtures were prepared accordingly. The PCR was performed with a total volume of 25 *μ*l containing a mixture of 2 *μ*l of template DNA, 2.5 *μ*l of 10x PCR buffer, 2.5 *μ*l (10 pmol/*μ*l) of each *mecA* gene forward and reverse primers, 0.5 *μ*l of dNTPS (10 mM), 1.5 *μ*l of MgCl_2_, and 0.5 *μ*l of Taq polymerase, and the remaining volume was filled by nuclease-free water to get a final volume of 25 *μ*l. PCR mixture without DNA template was used as a negative control. After preparation of mixtures, the PCR program was as follows: initial denaturation at 94°C for 5 min, 30 cycles of denaturation at 94°C for 60 s, annealing at 62°C for 30 s, extension at 72°C for 35 s, and final extension at 72°C for 10 min. Finally, the PCR products were stored at 4°C until analysis by agarose gel electrophoresis.

### 2.5. Agarose Gel Electrophoresis

Agarose gel electrophoresis was prepared with 1.5% agarose in 1x trice acetate EDTA (TAE) buffer, and 0.5 *μ*g/mL of ethidium bromide was added and mixed. A 12 *μ*l volume of PCR-amplified products was mixed with 3 *μ*l loading dye and then loaded into wells of agarose gel. Electrophoresis was carried out for 90 min (70 Volts/cm^2^) in 1x TAE buffer. DNA ladder (100 bp) was used to assess the PCR product size, then PCR products were visualized by UV light at 336 nm, and photographs were taken using a digital camera.

## 3. Results

A total of 1365 samples were cultured, and the isolation rate of *S. aureus* was 139/1365 (10.2%). Majority of the isolates were recovered from patients aged less than 5 years (51; 36.7%), followed by 16–30 years (42; 30.2%), 31–45 years (14; 10.1%), 6–15 years (13; 9.4%), and 46–60 years (13; 9.4%). The least number of isolates was recorded in age group greater than 60 years (6; 4.3%) (Table 1).

Most of the isolates were from blood (61 (43.9%)), followed by wounds (45 (32.4%)), urine (14 (10.1%)), discharges (11 (7.9%)), and body fluids (8 (5.8%)) (Table 2). Majority of the isolates were from the University of Gondar Comprehensive Specialized Hospital (53 (38.1%)), followed by Felege Hiwot Comprehensive Specialized Hospital (37 (26.6%)), Debre Markos Referral Hospital (29 (20.9%)), and Dessie Referral Hospital (20 (14.4%) (Table 2)).

Of the total 139 isolates collected from 4 different referral hospitals in Amhara region, 110 isolates were recovered by subculturing in the central Microbiology Laboratory at the University of Gondar. All these isolates were processed further and tested for 10 different antibiotics. High resistance rate was observed for penicillin (81; 73.6%), followed by cotrimoxazole (78; 70.9%), ceftriaxone (76; 69%), erythromycin (66; 60%), and tetracycline (65; 59.1%). However, relatively low resistance rates were observed for clindamycin (*n* = 24, 21.8%), gentamicin (34; 30.9%), and cefoxitin (38; 34.5%). Phenotypically, considering cefoxitin as surrogate marker, for methicillin resistance, 38 (34.5%) of the 110 *S. aureus* isolates were cefoxitin-resistant (Table 3) and thus classified as MRSA.

Among 110 isolates tested for 10 different commonly used antibiotics, 7 isolates were sensitive to all drugs tested and 23 isolates were resistant to one or two antibiotics. However, *S. aureus* isolates resistant to 3 or more antibiotic classes were 80 (72.7%) (Table 4).

For molecular detection of methicillin-resistant genes, out of 80 (72.7%) MDR isolates, we randomly selected 70 isolates of *S. aureus* and included from all study sites. Accordingly, we considered 40 isolates (13 were cefoxitin-resistant, 3 of them were intermediate, and 24 were sensitive) from the University of Gondar Comprehensive Specialized Hospital, 14 isolates (5 were cefoxitin-resistant, 2 of them were intermediate, and 7 were sensitive) from Felege Hiwot Comprehensive Specialized Hospital, 11 isolates (4 were cefoxitin-resistant, 3 of them were intermediate, and 4 were sensitive) from Dessie Referral Hospital, and 5 isolates (4 were cefoxitin-resistant and 1 was sensitive) from Debre Markose Referral Hospital (Table 5). In all cases, the isolates taken for *mecA* gene detection were phenotypically MDR.

**Figure 1:**
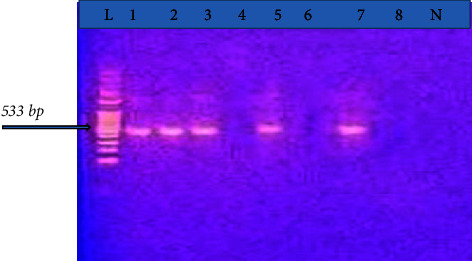
Agarose gel electrophoresis of the PCR amplification products of *S. aureus, mecA* gene (1.5% agarose, 70 V, 45 min.). L: the DNA molecular weight marker (100 bp ladder). Lanes 1, 2, 3, 5, and 7: positive PCR amplification of 533 bp for *mecA* gene. Lanes 4, 6, and 8: negative PCR amplification of 533 bp for *mecA* gene. N is a PCR product of negative control.

The PCR amplification result of the *mecA* gene was performed in all 70 clinical isolates of *S. aureus*. However, among the total of 70 isolates, *mecA* gene was detected only in 14 (20%) *S. aureus* isolates. The *mecA* gene positive in 14 isolates was phenotypically from MRSA and MSSA, but both of them were MDR (Figure 1). Although its distribution is different, *mecA* gene producing methicillin-resistant*S. aureus* was reported in all study sites.

## 4. Discussions

*S. aureus* is a main pathogenic bacterium which causes severe human health problems globally [16], and its antimicrobial resistance characteristics have made it more rebellious in the health institutions [17].

The isolation rate of *S. aureus* in the current study was 139/1365 (10.2%) which is lower than a study conducted in Ethiopia (79/94 (84.0%)) [18] and Nigeria (55/360 (15.3%)), and the occurrence of *S. aureus* was the highest in wound swabs [19], but in the present study, the highest isolates were recovered from blood sample followed by wound specimen.

Majority of the isolates were recovered from patients aged less than 5 years (51 (36.7%)), followed by 16–30 years (42 (30.2%)), while the least number of isolates was from patients greater than 60 years. This is in line with an observation from previous Ethiopian report where the rate of isolation of *S. aureus* was higher in lower age (15–24 years) (46/210 (21.9%)) [20] and from Eritrean study where it was significantly associated with lower age, 13 to 18 years (78.6%) and <13 years old (85.0%), and lower rate of isolation was recorded in older age (≥61 years old) [21].

The most common clinical specimen for *S. aureus* isolates in the current study was blood (61 (43.9%)), followed by wounds (45 (32.4)). However, the previous study conducted in Ethiopia demonstrated that the highest rate of isolation was observed in pus (118/213 (55.4%)), followed by nasal swab (9/27 (33.3%)) [20]; in Eritrea, highest isolates (64/103, 62.1%) were obtained from pus specimens examined, followed by blood specimens (6/15 (40.0%)) [21]. The highest prevalence of *S aureus* was also observed from seminal fluid of patients (9/36 (25%)), followed by wound swabs (13/87 (15%)) while the lowest (5.4%) was found from urine in a study from Nigeria [17]. Another study conducted in Nigeria also revealed that the occurrence of *S. aureus* was highest in wound swabs, vaginal swabs, and urine [19]. The Iranian report on distribution analysis of the *S. aureus* isolates among clinical samples showed that most of the isolates (29.0%) were recovered from the pus and the lowest (1.4%) was found from cerebrospinal fluid [15]. The variations in occurrence of the organism in the different clinical samples across many studies show the versatility of this organism amongst other bacteria which makes it the most endemic pathogen in clinical settings, and it may likely be responsible for various infections such as UTI, wound infection, deep tissue infections, including osteomyelitis, arthritis, endocarditis, and cerebral, pulmonary, renal, and breast abscesses [22].

In the present study, the antimicrobial resistance rates of 110 *S. aureus* isolates against 10 antibiotics were 73.6%, 70.9%, 69%, 60%, 59.1%, 35.5%, 35.5%, 34.5%, 30.9%, and 21.8% toward penicillin, cotrimoxazole, ceftriaxone, erythromycin, tetracycline, chloramphenicol, ciprofloxacin, cefoxitin, gentamicin, and clindamycin, respectively. These findings are almost in parallel with a study conducted in Ethiopia where the isolates were resistant to ampicillin (100%), cefoxitin (68.4%), clindamycin (63.3%), cephalothin (59.5%), tetracycline (57%), cotrimoxazole and bacitracin (53.2%, each), and erythromycin (51.9%) [18], and in Iran where the percentage of resistance of *S. aureus* was 100%, 59.1%, 57.7%, 50%, 49.1%, 48.3%, 47.6% and 47.6%, 25%, and 0.7% to penicillin, tetracycline, ciprofloxacin, erythromycin, gentamicin, cotrimoxazole, cephalothin, and oxacillin, clindamycin, and vancomycin, respectively [15]. The highest level of antimicrobial resistant *S. aureus* in a Nigerian study was 68% to ceftazidime, followed by cloxacillin (48%) while the least resistance (26%) was observed for meropenem [17]. In line with the current study, another study from Nigeria also demonstrated that the isolates from three hospitals were highly (≥50%) resistant to all the antibiotics tested (ampicillin, ciprofloxacin, erythromycin, oxacillin, rifampicin, clindamycin, sulphamethoxazole/trimethoprim, and streptomycin), but moderately (≤40%) resistant to gentamicin and levofloxacin [19]. This variation might be attributed to differences in patients' hospital stay, level of infection control practices by health facilities, previous exposure of patients to antibiotics, and irrational use of antibiotics.

Phenotypically, considering cefoxitin as surrogate marker for methicillin test, 38 (34.5%) of the isolates of *S. aureus* were methicillin-resistant in the current study which is in agreement with the pooled prevalence of MRSA reported in Ethiopia (32.5%) [23]. However, the current finding of MRSA is lower than a report from Ethiopia, where 54 (68.4%) of the isolates were MRSA [18]; from Eritrea, 59 (72.0%) of the isolates were MRSA [21]; from Nigerian studies, 44.0% [17]; and 40.4% of the isolates were MRSA [24]; and from Iran, 133/279 (47.6%) of the isolates were MRSA [15]. On the other hand, the present report is higher than another previous report from Ethiopia where 34/194 (17.5%) of the *S. aureus* isolates were found to be MRSA [20]; in Iraq, the prevalence of MRSA was 114/429 (26.54%) [25]. The possible explanation for the observed discrepancies across the literature might be associated with the variation of the methods used to detect methicillin resistance. Some studies used cefoxitin and others used oxacillin as a surrogate marker for the detection of methicillin resistance.

The MDR isolates observed in the current study was 80/110 (72.7%) which is in line with a previous report in Ethiopia (65 (82.3%)) [18]. However, the MDR *S. aureus* observed in the present study is higher than a previous study reported from Ethiopia where 98 (50.5%) of the *S. aureus* isolates were MDR [20], from Eritrea where 17/43 (39.5%) isolates were MDR [21]; and from Saudi Arabia where 47% of MRSA were MDR [26].

The PCR amplification result of *mecA* gene, a gene that confers resistance to methicillin and most *β*-lactam antibiotics, was obtained in 70 clinical isolates of *S. aureus*. However, among the total of 70 isolates, *mecA* gene was detected only in 14 (20.0%) *S. aureus* isolates with an amplicon of 533 bp considered as indicative with the presence of *mecA* gene. Although its distribution is different, *mecA* gene producing MRSA was reported in all study sites. This is similar with a study from Nigeria that phenotypic resistance to cefoxitin was 46.5%, while the *mecA* gene was 19.2% [24]. Another study from Nigeria indicated that *S. aureus* isolates with phenotypic resistance to methicillin (oxacillin) were tested for *mecA* gene and none of the isolates contained the *mecA* gene [27]. Nwaogaraku et al. from Nigeria showed that all isolates of MRSA from blood samples of pigs were *mecA* negative on PCR [28]. However, the present study is different from many studies performed elsewhere [26, 29, 30]. The possible explanation why phenotypically MRSA-positive isolates did not show *mecA* gene might be due to loss of the *mecA* gene during prolonged storage [31] or other mechanisms other than the presence of *mec*A gene (*mecC* and *mecB*) responsible for methicillin-resistant*Staphylococcus aureus* [32, 33].

## 5. Conclusion and Recommendation

Phenotypic resistance to cefoxitin was 34.5%. This prevalence overestimated the prevalence of MRSA, as the *mecA* gene that encodes resistance to methicillin was detected by PCR in 20.0% of the *S. aureus* isolates. A large-scale study for *mecA* gene detection is important to re-assure the discrepancy between phenotypic and *mecA* gene detection in methicillin-resistant S. *aureus*.

## Figures and Tables

**Table 1:** Distribution of *S. aureus* isolates in different clinical samples with respect to age from four referral hospitals of Amhara region, Ethiopia (2017-2018).

Age category	Clinical samples (%)					Total
	Urine	Blood	Wound	Discharges	Body fluids	
≤5 years	2 (1.4)	33 (23.7)	12 (8.6)	2 (1.4)	2 (1.4)	51 (36.7)
6–15 years	—	7 (5.0)	5 (3.6)	1 (0.7)	—	13 (9.4)
16–30 years	9 (9.5)	15 (10.8)	15 (10.8)	2 (1.4)	1 (0.7)	42 (30.2)
31–45 years	—	2 (1.4)	6 (4.3)	5 (3.6)	1 (0.7)	14 (10.1)
46–60 years	2 (1.4)	1 (0.7)	6 (4.3)	1 (0.7)	3 (2.2)	13 (9.4)
>60 years	1 (0.7)	3 (2.2)	1 (0.7)	—	1 (0.7)	6 (4.3)
Total	14 (10.1)	61 (43.9)	45 (32.4)	11 (7.9)	8 (5.8)	139 (100)

**Table 2:** Distribution of *S. aureus* isolates from four referral hospitals of Amhara region, Ethiopia (2017-2018).

Isolates	Name of referral hospitals	Clinical samples					
		Urine	Blood	Wound	Discharges^*∗*^	Body fluids	Total
*S. aureus*	University of Gondar Comprehensive Specialized Hospital	2 (3.8)	14 (26.4)	29 (54.7)	2 (3.8)	6 (11.3)	53 (100)
	Felege Hiwot Comprehensive Specialized Hospital	2 (5.4)	23 (62.2)	10 (27.0)	2 (5.4)	—	37 (100)
	Dessie Referral Hospital	8 (40.0)	1 (5.0)	3 (15.0)	7 (35.0)	1 (5.0)	20 (100)
	Debre Markos Referral Hospital	2 (6.9)	23 (79.3)	3 (10.3)	—	1 (3.4)	29 (100)
Total		14 (10.1)	61 (43.9)	45 (32.4)	11 (7.9)	8 (5.8)	139 (100)

^*∗*^Eye and ear discharges.

**Table 3:** Drug resistance patterns of *S. aureus* against commonly used antibiotics from referral hospitals of Amhara region (2017-2018).

Bacterial isolates		ERY	PEN	CLI	SXT	TET	CIP	CHL	GEN	CRO	FOX
*S. aureus*	S	37 (33.6)	25 (22.7)	86 (78.2)	31 (28.2)	42 (38.2)	65 (59.1)	69 (62.7)	70 (63.6)	29 (26.4)	64 (58.2)
	I	7 (6.4)	4 (3.6)	—	1 (0.9)	3 (12.7)	6 (5.5)	2 (1.8)	6 (5.5)	5 (4.5)	8 (7.3)
	R	66 (60)	81 (73.6)	24 (21.8)	78 (70.9)	65 (59.1)	39 (35.5)	39 (35.5)	34 (30.9)	76 (69.1)	38 (34.5)

ERY = erythromycin; PEN = penicillin; CLI = clindamycin; SXT = cotrimoxazole; TET = tetracycline; CIP = ciprofloxacin; CHL = chloramphenicol; GEN = gentamycin; CRO = ceftriaxone; FOX = cefoxitin; S = sensitive; I = intermediate; R = resistant.

**Table 4:** Resistance profiles of 110 *S. aureus* isolates from clinical samples at the four referral hospitals of Amhara region (2017-2018).

Antibiogram pattern	Number of *S. aureus* isolates
All drug sensitive	7
CHL (not MDR)	2
TET (not MDR)	1
SXT (not MDR)	4
FOX (not MDR)	2
TET, GEN (not MDR)	1
PEN, SXT (not MDR)	1
PEN, CRO (not MDR)	1
PEN, CHL (not MDR)	1
PEN, CLI (not MDR)	1
SXT, CRO (not MDR)	1
ERY, PEN (not MDR)	1
ERY, PEN, CRO (not MDR)	2
PEN, SXT, CRO (not MDR)	2
PEN, SXT, CRO, FOX (not MDR)	1
PEN, CHL, CRO, FOX (not MDR)	1
PEN, TET, CRO, FOX (not MDR)	1
SXT, TET, CRO (MDR)	1
PEN, SXT, TET, CRO (MDR)	1
Other isolates resistant to 3 or more antibiotic classes	78
Total non-MDR isolates	30 (27.3%)
Total MDR isolates	80 (72.7%)
Total	110 (100%)

ERY = erythromycin; PEN = penicillin; CLI = clindamycin; SXT = cotrimoxazole; TET = tetracycline; CIP = ciprofloxacin; CHL = chloramphenicol; GEN = gentamycin; CRO = ceftriaxone; FOX = cefoxitin. MDR = *S. aureus* isolates resistant to 3 or more antibiotic classes.

**Table 5:** PCR results for *mecA* gene for methicillin-resistant*S. aureus* from selected isolates at different referral hospitals of Amhara region, Ethiopia.

Study sites	Total isolates subjected for PCR	*mecA-*negative	*mecA-*positive
University of Gondar Comprehensive Specialized Hospital	40 (100)	29 (72.5)	11 (27.5)
Felege Hiwot Comprehensive Specialized Hospital	14 (100)	13 (92.9)	1 (7.1)
Dessie Referral Hospital	11 (100)	10 (90.9)	1 (9.1)
Debre Markose Referral Hospital	5 (100)	4 (80.0)	1 (20.0)
Total	70 (100)	56 (80.0)	14 (20.0)

## Data Availability

The data used and analyzed during the current study are available from the corresponding author on reasonable request.
